# Terrigenous dissolved organic matter persists in the energy-limited deep groundwaters of the Fennoscandian Shield

**DOI:** 10.1038/s41467-022-32457-z

**Published:** 2022-08-17

**Authors:** Helena Osterholz, Stephanie Turner, Linda J. Alakangas, Eva-Lena Tullborg, Thorsten Dittmar, Birgitta E. Kalinowski, Mark Dopson

**Affiliations:** 1 Marine Chemistry, Leibniz Institute for Baltic Sea Research Warnemünde, Rostock, Germany; 2 Ecology and Evolution in Microbial model Systems (EEMiS), Linnaeus University, Kalmar, Sweden; 3 Department of Forest Mycology and Plant Pathology, Swedish University of Agricultural Sciences, Uppsala, Sweden; 4 Swedish Nuclear Fuel and Waste Management Company, Äspö Hard Rock Laboratory, Oskarshamn, Sweden; 5 Terralogica AB, Gråbo, Sweden; 6 Marine Geochemistry, Institute for Chemistry and Biology of the Marine Environment, Carl von Ossietzky University, Oldenburg, Germany; 7 Helmholtz Institute for Functional Marine Biodiversity, Carl von Ossietzky University, Oldenburg, Germany

**Keywords:** Carbon cycle, Water microbiology

## Abstract

The deep terrestrial biosphere encompasses the life below the photosynthesis-fueled surface that perseveres in typically nutrient and energy depleted anoxic groundwaters. The composition and cycling of this vast dissolved organic matter (DOM) reservoir relevant to the global carbon cycle remains to be deciphered. Here we show that recent Baltic Sea-influenced to ancient pre-Holocene saline Fennoscandian Shield deep bedrock fracture waters carried DOM with a strong terrigenous signature and varying contributions from abiotic and biotic processes. Removal of easily degraded carbon at the surface-to-groundwater transition and corresponding microbial community assembly processes likely resulted in the highly similar DOM signatures across the notably different water types that selected for a core microbiome. In combination with the aliphatic character, depleted δ^13^C signatures in DOM indicated recent microbial production in the oldest, saline groundwater. Our study revealed the persistence of terrestrially-sourced carbon in severely energy limited deep continental groundwaters supporting deep microbial life.

## Abstract (web summary)

Dissolved organic matter in the Fennoscandian Shield deep continental bedrock fracture waters of varying characteristics and ages carries a strong terrigenous signature, and only a small proportion of this potential energy source links to the deep biosphere microbial community.

## Introduction

Groundwater is the earth’s largest active source of freshwater^1^ containing a vast store of poorly characterized dissolved organic matter (DOM)^2^, an actively cycled key component in the global carbon cycle. As the water moves into deeper layers, nutrients and available organic carbon are removed, severely limiting the energy supply to the deep biosphere life estimated to contain 2–6 × 10^29^ cells^3^ that is approximately one quarter of the global microbial biomass^4^. Key processes in deep subsurface carbon cycling depend on the recalcitrance of the organic matter to microbial utilization^5^, input of metabolic oxidants and reductants, and aquifer permeability^6^. While acting as a nutrient and energy source for heterotrophic life^7^, the composition and concentration of the DOM also impacts trace element and contaminant transport^8,9^, especially in anthropogenically impacted groundwater systems. Climate change and increasing urbanization exert pressures on the deep groundwater systems affecting DOM input, storage, and turnover^2,10^. Yet to date, few studies have addressed DOM composition and its associated bioavailability in deep terrestrial groundwaters.

Plant litter and soil comprise the main sources of DOM in shallow groundwaters^11^. Plant-derived molecular constituents and a high overlap with riverine fulvic acids, carrying a large number of preferentially biodegraded nitrogen- and sulfur-containing compounds, were attributed to groundwater DOM at depths <3 m^12,13^. Sulfur-containing organic matter can be a dominant component of rainwater^14^, but can also be released by microbes or abiotically produced in anoxic settings, enhancing the recalcitrance of the organic matter^15–17^. Permeating through soils and sediments, selective interactions of the organic matter occur with microbes, minerals, and water that integrated in the “regional chromatography” effect shape a homogenized DOM signature in the saturated zone^18,19^. Nevertheless, the source and history of groundwater DOM remain imprinted in its composition, with bulk organic carbon and isotopic compositions similar to surface soils indicating a surface origin and vertical transport into aquifer systems^20^ that varies seasonally^21,22^. The proximity and connectivity to organic-rich layers drive microbial abundance and activity as well as microbial community composition^23,24^ and DOM from chemolithoautotrophic communities in deep basaltic or granitic intrusions may be important as the long timescales increase the proportion of microbial-derived and transformed DOM over allochthonous, terrigenous material in the oligotrophic systems^25–27^.

The Äspö Hard Rock Laboratory (HRL) on the Swedish Baltic Sea coast facilitates access to deep continental groundwaters in the Fennoscandian Shield. Its 3.6 km long tunnel partially extends below the Baltic Sea at depths down to 460 meters below sea level (mbsl). The groundwater components consist of old saline water, brackish water from the overlying Baltic Sea and its predecessors such as the Littorina Sea, and fresh waters from temperate and cold climate (glacial meltwaters) of the Pleistocene^28–30^. The present groundwaters broadly fall into four main types according to water chemistry signatures: marine, meteoric, old saline, and glacial^30^. While the brackish Baltic Sea water is classified as a marine source, its DOM is of predominantly terrestrial origin, i.e., comprising about 75% in the Baltic Proper^31^. During the 30 years since the Äspö HRL tunnels were constructed, complex mixing has occurred where some fracture zones facilitate downward percolation of Baltic Sea water whereas other fractures promote upward flux of deep saline waters. The Fennoscandian Shield microbial community has a core microbiome between the different groundwater types often comprised of small cells with streamlined genome sizes that likely represent adaptations to an oligotrophic lifestyle^32–34^. While isotopically light carbon indicates slow, but persistent microbial transformation of surface-derived DOM^35^, the link between the DOM reservoir and the microbial community is largely unknown.

**Fig. 1:**
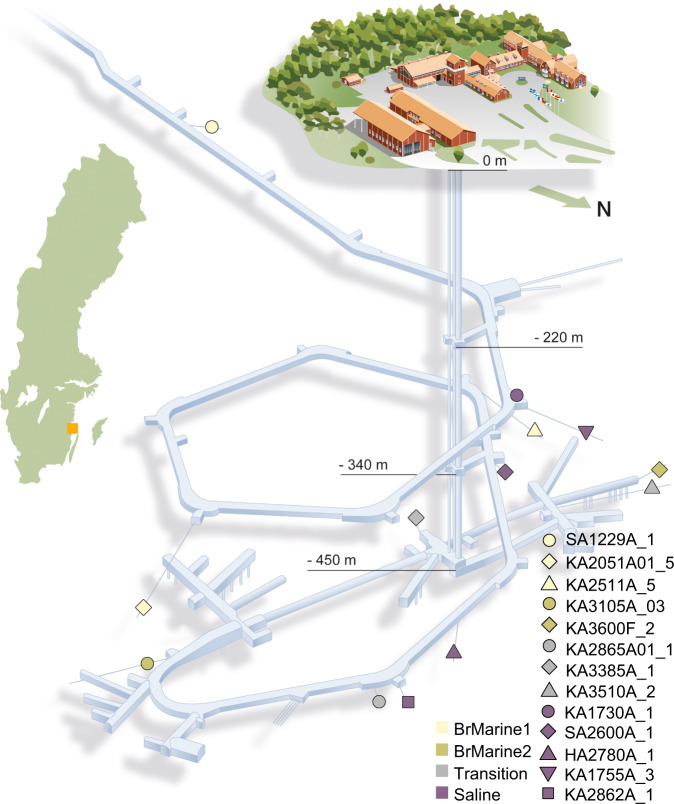
The Äspö HRL tunnel system and sampled boreholes. Location of the Äspö HRL in Kalmar County, Sweden, is shown in the schematic map; depth, location and water type of samples included in this study are indicated on the tunnel system reproduction. A portion of the tunnel (including boreholes SA1229A_1, KA2051A01_5, and KA3105A_03) extends below the Baltic Sea. Figure reprinted and modified from ref. 94 with permission from Elsevier.

In this study, DOM concentration and molecular composition in conjunction with stable and radiogenic carbon and water isotopic investigations, water chemistry, and microbial community structure in Äspö HRL fracture waters (Fig. 1) of different depths, ages, and sources were analyzed to disentangle mechanisms behind organic matter cycling in the deep biosphere. We include a saline pre-Holocene groundwater, potentially representative of often overlooked natural saline groundwaters worldwide^36^. We hypothesized that terrestrial surface DOM fuels microbial life in the deep continental bedrock fractures. In addition, we suggest that abiotic transformations occurring on long timescales and microbial reworking of the aged DOM is likely in the low carbon and energy groundwaters with cycling of sulfur compounds playing an important role.

## Results

### Type and origin of the groundwaters

**Fig. 2:**
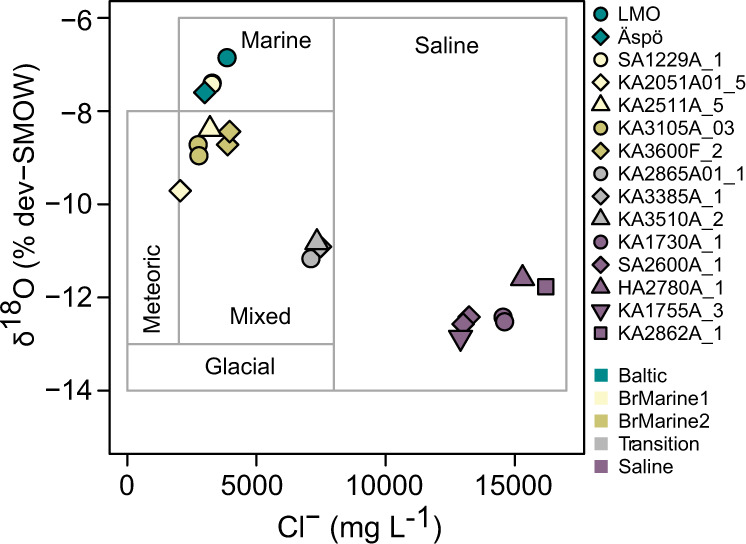
Hydrochemical classification according to Cl^−^ and δ^18^O. Boxes denote origin of water according to the model presented by ref. 30.

Using chloride concentrations, δ^18^O values, and Cl/Mg plus Br/Cl ratios to distinguish water origin in the aquifer at Äspö HRL^28,30^ grouped the samples (Table 1 & Fig. 2) into: (i) ‘Baltic Sea’ water with typical salinities and δ^18^O values for these brackish waters^30,37^; (ii) ‘BrMarine1’ recent brackish-marine waters intruded into the boreholes; (iii) ‘BrMarine2’ Holocene marine waters older than present day Baltic Sea; (iv) ‘Transition’ waters that carry signatures from both BrMarine and old saline waters; and (v) brine-type old ‘Saline’ water of unknown origin with residence times in the bedrock of ≥100,000 years^38^ mixed to various degrees with old meteoric water of cold climate origin, likely glacial and pre-Holocene. The origin of the brine component is complex and may involve leaching of marine sediments (e.g., Palaeozoic evaporites) later influenced by water rock interaction through time^38^. The Baltic Sea samples clustered with BrMarine1 borehole SA1229A that accessed a large section with high connectivity to the Baltic Sea. All other BrMarine1 and BrMarine2 groundwaters grouped within the area of mixed water origin, with one borehole sample (KA2051A01_5, BrMarine1) moved toward meteoric characteristics. KA2865A01_1 and KA3385A_1 waters associated closest with KA3510A_2 classified as Transition-type waters of intermediate Cl/Mg ratios.

**Table 1:** Water chemistry parameters of Baltic Sea water and borehole samples

Borehole/Sampling Area	Water type (this study)	Water type (Lopez-Fernandez et al. 2018)^a^	Date	Mean depth	Section length	*n*	Conductivity	pH	Alkalinity	Ca	Mg	Cl	SO_4_^2−^	DOC	TDN	Extraction efficiency
			(mm-yy)	m	m		mS m^−1^		mg L^−1^	mg L^−1^	mg L^−1^	mg L^−1^	mg L^−1^	mg L^−1^	mg L^−1^	% DOC
**Baltic Sea water**																
LMO	Baltic Sea		04–19			2	1230	7.98	98.9	102	245	3860	515	3.79 ± 0.07	0.24 ± 0.01	63
Äspö	Baltic Sea		04–19			2	980	7.32	75.4	82.9	190	3000	435	7.05 ± 0.06	0.43 ± 0.01	68
**Äspö HRL groundwaters**																
SA1229A_1	BrMarine1	MM-171.3	11–18	171	19.6	1	1031	7.25	252.6	334	146	3282	266	5.81 ± 0.21	5.62 ± 0.16	74
			04–19		19.6	1	1030	7.28	249	345	140	3270	259	5.84 ± 0.07	5.52 ± 0.10	75
KA2051A01_5	BrMarine1	MM-349.1	03–19	349	15.0	2	677	7.69	155	368	45.7	2050	181	6.20 ± 0.09	0.33 ± 0.01	79
KA2511A_5	BrMarine1	MM-349.5	03–19	395	7.0	2	1000	7.54	154	471	81.3	3200	300	4.47 ± 0.04	0.69 ± 0.01	76
KA3105A_3	BrMarine2	MM-415.6	11–18	416	2.0	1	871	7.50	127.6	455	72.3	2747	274	4.58 ± 0.13	0.38 ± 0.00	78
			03–19		2.0	1	868	7.72	121	463	69	2770	261	4.44 ± 0.05	0.38 ± 0.02	82
KA3600F_2	BrMarine2	MM-446.8	11–18	447	1.5	1	1197	7.47	126.6	708	75.2	3884	299	3.97 ± 0.03	0.45 ± 0.02	79
			03–19		1.5	1	1190	7.49	126	714	72.6	3960	293	3.85 ± 0.03	0.44 ± 0.03	78
KA2865A01_1	Transition	NA^b^	03–19	380	27.7	2	1960	7.38	78.7	2090	56.3	7110	350	2.94 ± 0.02	0.29 ± 0.02	78
KA3385A_1	Transition	TM-448.4	03–19	448	2.1	2	2060	7.48	25.1	2220	55.4	7480	399	1.17 ± 0.02	0.13 ± 0.01	87
KA3510A_2	Transition	NA^b^	03–19	507	14.0	2	2060	7.75	11.5	2280	32.4	7340	488	1.41 ± 0.04	0.10 ± 0.01	81
SA2600A_1	Saline	OS-345.0	11–18	345	18.1	1	3404	7.53	16.4	4400	33.7	13230	596	0.90 ± 0.05	0.11 ± 0.02	87
			03–19		18.1	1	3410	7.50	16.6	4620	32.1	13000	606	0.95 ± 0.04	0.10 ± 0.01	82
HA2780A_1	Saline	NA^b^	03–19	381	42.1	2	3860	7.81	9.1	5590	32.8	15300	644	0.85 ± 0.04	0.08 ± 0.01	83
SA1730A_1	Saline	OS-237.0	11–18	237	18.7	1	3700	7.66	12.6	5050	31.6	14540	615	0.78 ± 0.01	0.09 ± 0.01	88
			03–19	237	18.7	1	3640	7.60	15.8	5100	29.4	14600	623	0.98 ± 0.01	0.09 ± 0.01	82
KA1755A_3	Saline	OS-279.9	03–19	280	72.0	2	3380	7.61	9.3	4790	30.5	12900	592	0.76 ± 0.04	0.08 ± 0.02	79
KA2862A_1	Saline	OS-380.6	03–19	380	16.0	2	4140	7.65	8.5	6120	33	16200	595	0.92 ± 0.04	0.08 ± 0.01	82

Sampling and borehole details, water chemistry, and DOC and TDN concentrations as well as solid-phase extraction efficiency on a carbon basis.

^a^The second water type designation is provided for comparison with other studies of the Äspö HRL (MM modern marine, TM thoroughly mixed, OS old saline).

^b^NA not available. For additional parameters, see Table S1.

### Dissolved organic matter in fracture waters

DOC concentrations decreased with increasing conductivity and were below 1.15 mg L^−1^ in all Saline waters while the Linnaeus Microbial Observatory (LMO) and near Äspö Island Baltic Sea values framed the BrMarine samples (Table 1). Dissolved organic nitrogen (DON) concentrations were higher in waters of lower conductivity. With the exception of borehole SA1229A_1 that had >5 mg L^−1^ total dissolved nitrogen (TDN) mostly as ammonium of an unknown source, dissolved C/N ratios were not significantly different between water types at an average of 19.4 ± 6.1.

Solid-phase carbon extraction efficiencies were 81 ± 4% and 66 ± 4% across all deep fracture waters and the two Baltic Sea samples, respectively (Table 1). The DOM composition comprised 12,728 monoisotopic molecular formulas (MFs) assignments between 35 samples (average 7961 ± 464; Table S1) in the 96–1000 Da mass range from FT-ICR-MS analysis. Of these, 3772 MFs were found in all samples and accounted for on average 47 ± 3% of the MFs by count and 85 ± 2% of the relative intensities per sample. 309 MFs (0.23 ± 0.13% of relative intensities) were unique to the Saline-type DOM that plotted at high H/C and low O/C ratios in van Krevelen space used to visualize tentative structural characteristics of MFs^39^, including few O-poor aromatics and mostly highly unsaturated O-poor or unsaturated MFs, some containing N (Supplementary Fig. S1). 110 MFs (0.21 ± 0.04% of relative intensities) were unique to the two Baltic Sea samples that plotted in the region of tannin-like and condensed aromatic MFs of terrigenous origin. The unique MFs of the BrMarine samples (*n* = 438; 0.26 ± 0.11% relative intensity) were widely spread across van Krevelen space besides the area of O-rich aromatics and highly unsaturated MFs.

**Fig. 3:**
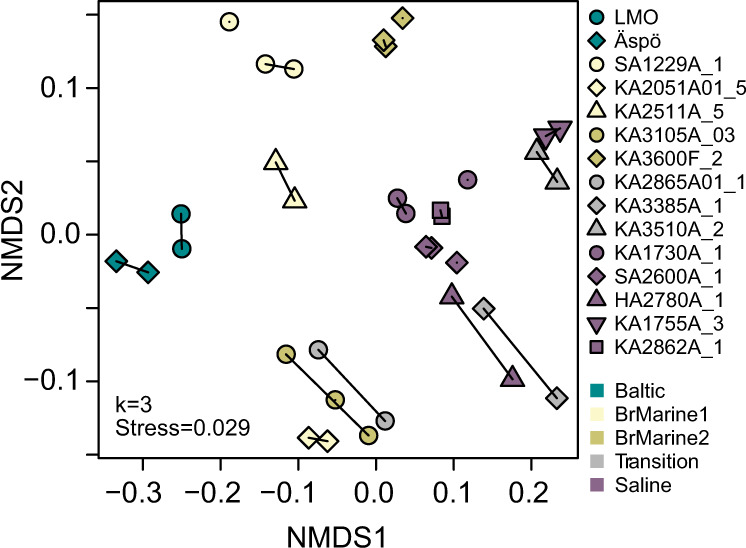
NMDS of DOM molecular composition. Shapes denote boreholes and colors show water types. Lines connect batch 2 replicates.

A gradient from Baltic Sea, through Transition, to Saline groundwater origins was partially reflected by the distribution of DOM compositions along the first axis of NMDS ordination (Fig. 3). Hydrochemical composition based on eight main parameters (Na, K, Ca, Mg, δ^18^O, δ^2^H, SO_4_^2−^, D, and Cl^−^) similar to those used previously for water type classification at Äspö HRL^29^ and DOM composition were significantly related (distance-based redundancy analysis, db-RDA), 9999 permutations, *n* = 15, *F* = 3.6, *p* < 0.01). Overall, hydrochemistry explained about 83% of DOM variability with some differences in classifications remaining. BrMarine1 samples KA2511A_5 and SA1229A_1 grouped closest with the Baltic Sea waters, while KA2051A01_5 associated with BrMarine2 and Transition boreholes KA3105A_3 and KA2865A01_1. The Transition DOM formed a tight group in the hydrochemical classification, but their mixed origin stood out in DOM molecular space. The Transition-type waters KA3510A_2 and KA3385A_1 grouped closest to Saline KA1755A_3 and HA2780A_1, respectively, while KA2865A01_1 more closely affiliated with BrMarine2 borehole KA3105A_3.

**Fig. 4:**
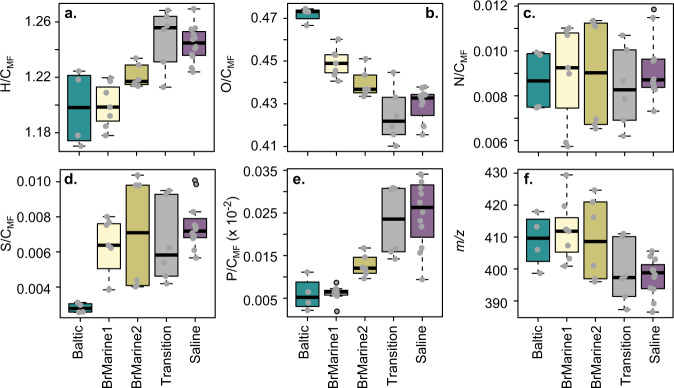
DOM elemental composition. Elemental ratios H/C_MF_ (**a**), O/C_MF_ (**b**), N/C_MF_ (**c**), S/C_MF_ (**d**), P/C_MF_ (**e**), and m/z (**f**) by water type. Boxplots display minimum, first quartile, median, third quartile, maximum, and outliers (black circles), including technical and analytical replicates (*n* = 35, gray dots) colored by water type.

**Fig. 5:**
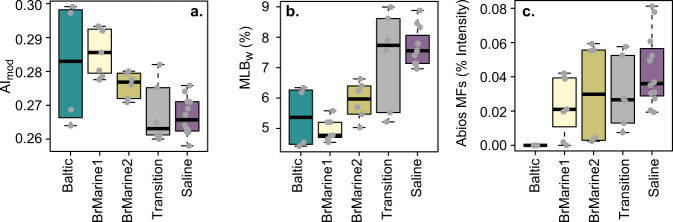
DOM composition indices. Aromaticity from aromaticity index AI_mod_ (**a**), lability from MLB_w_ (**b**), and percentage contribution of AbioS MFs to total sample intensity by water type (**c**). Boxplots display minimum, first quartile, median, third quartile, maximum, and outliers (black circles), including technical and analytical replicates (*n* = 35, gray dots) colored by water type.

The Baltic and BrMarine1 waters showed similar H/C_MF_ and P/C_MF_ ratios (weighted means from MF assignments) that were low compared to older groundwater samples (Fig. 4). Although the BrMarine group DOM composition was not coherent, its average mass tended to be higher than that of Transition- and Saline-type DOM. The N/C_MF_ ratio varied across all water types while the O/C_MF_ ratio and S/C_MF_ ratios were most different for Baltic Sea DOM. Sulfate and sulfide concentrations negatively related in BrMarine1, BrMarine2, and KA2865A01_1, denoting ongoing sulfate reduction in the most recent waters. The DOM S/C_MF_ ratio strongly correlated with the relative contribution of AbioS^40^ peaks, indicative of abiotic sulfurization under anoxic conditions, of which 13 (out of 15) were detected in this dataset (*R* = 0.94, *p* < 0.001). The AbioS peaks were not present in Baltic Sea water and barely present in KA2051A01_5 (BrMarine1) and KA3105A_3 (BrMarine2). In addition, the S/C_MF_ ratio was highly correlated to the number of S-containing MFs (*R* = 0.95, *p* < 0.001), implying the production of new S-containing MFs rather than addition to already existing ones. The presumed lability of the DOM based on the lability index MLB_w_ was highest in the oldest, Saline- and some Transition-type waters (Fig. 5). The aromaticity, assessed via the modified aromaticity index AI_mod_, covered a wide range between the Baltic Sea samples closest to the coast that decreased toward Transition and Saline DOM.

**Fig. 6:**
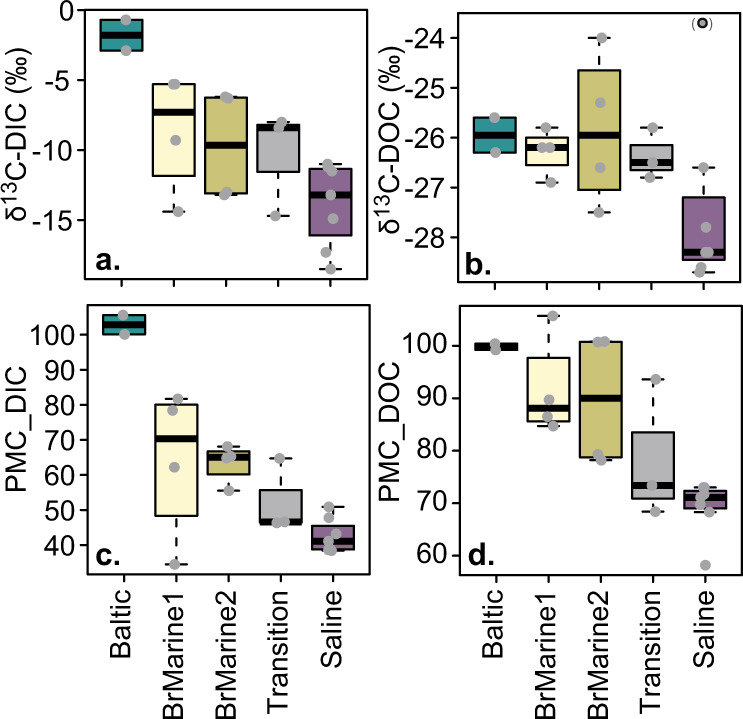
Carbon isotopic composition. Stable (**a**, **b**) and radiogenic (**c**, **d**) isotopic compositions of inorganic and organic carbon. Technical and analytical replicates are included (gray dots). Boxplots display minimum, first quartile, median, third quartile, maximum, and outliers (black circles), including technical and analytical replicates (*n* = 15, gray dots) colored by water type.

The radiocarbon age of the bulk inorganic and organic carbon fractions was assessed as percent modern carbon (pMC_DOC, pMC_DIC; Fig. 6) with pMC values around 100 corresponding to recent carbon and 60 corresponding to a mean residence time of around 4200 years. pMC_DOC ranged from 105.7 in borehole KA2051A01_5 (BrMarine2) to 58.2 in borehole KA1755A_3 (Saline), but most old Saline waters had a DOC radiocarbon age of around 69 pMC corresponding to ~3000 years. pMC_DIC also strongly decreased from Baltic Sea (105.6 near Äspö Island) and BrMarine groundwaters compared to values of 40–50 in most Saline waters, a Transition-type water (KA3510_2), and the BrMarine2 water from borehole KA2511A_5. pMC_DOC and pMC_DIC were significantly positively correlated (*R* = 0.66, *p* < 0.001) but the organic fraction showed a higher pMC value than the inorganic fraction, except for the Baltic Sea waters, that was mainly attributed to dissolution of calcite within the overburden or in the uppermost fractures. The relatively young age of the inorganic and organic carbon in the Saline-type fracture waters was surprising regarding their implicit origin. The saline component endmember (45,000 mg Cl L^−1^) has a long residence time manifested in Mg/Cl, Br/Cl ratios, He content, and δ^18^O/δH ratio^38^. However, the Cl^−^ concentrations were below 15,000 mg L^−1^ with the dilute components being of glacial origin or possibly an older meteoric water. Introduction of modern water portions or contamination through e.g., biofilm formation in the sampling system is unlikely as sampling section and tubing were flushed with several volumes of fracture water before each sample was taken, similar carbon extraction efficiencies were found over all section and tubing lengths, and FT-ICR-MS spectra showed no obvious contaminants. The detected trends between water types thus likely hold true but nevertheless, these potential caveats need to be considered especially for the low-DOC Saline-type water.

δ^13^C-DIC values ranged from −0.7‰ in Baltic Sea water to −18.5‰ in Saline groundwaters while δ^13^C-DOC values ranged from −24.0 to −28.7, ‰ respectively (Fig. 6) except for one sample from borehole SA2600A_1 that showed very different values between batch 1 (δ^13^C-DOC = −28.6‰) and batch 2 (δ^13^C = −23.70‰). As all other samples were consistent between the years and the second sampling of SA2600A_1 was not otherwise conspicuous, this value was disregarded during further interpretations. δ^13^C values of organic and inorganic carbon were positively related (*R* = 0.70, *p* < 0.01) with Δ^13^C_DIC−DOC_ values of −16.6 ± 4.1‰ for all samples and −14.1 ± 3.3‰ for old saline waters. The solid-phase extracted DOC stable carbon isotope signature (δ^13^C-SPE-DOC) did not mirror the full extent of δ^13^C-DOC and ranged from −26.1 to −28.4‰ (*R* = 0.42, *p* < 0.05). The agreement between δ^13^C-SPE-DOC and δ^13^C-DOC values was good, but deviation was highest for Baltic Sea water with the lowest extraction efficiencies (Supplementary Fig. S2). On DOM molecular level, the δ^13^C values were most strongly correlated to the MF fraction assigned to the aromatic, oxygen-rich molecular class (*R* = 0.70, *p* < 0.01) that comprised between 4 and 9% of the relative intensity.

### Microbial communities and DOM

**Fig. 7:**
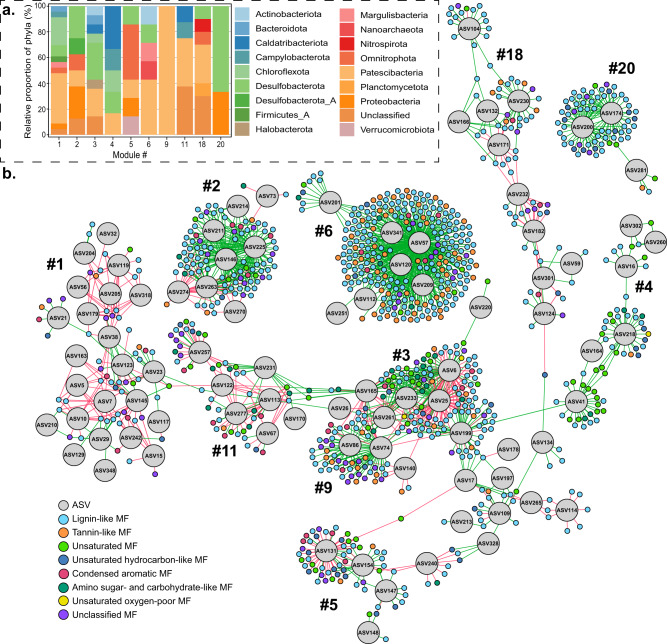
Network showing the links between MFs and microbial ASVs. Bar plot of taxonomic affiliation (phylum level) of ASV nodes of the ten modules (**a**). Network of network ASVs and MFs with positive and negative links represented by green and red edges, respectively (**b**). Node colors illustrate ASVs (gray) and compound classes for the MFs. Numbers represent modules. Details on ASVs can be found in Supplementary Data 1.

The microbial communities in Äspö HRL groundwaters were dominated by 16 S rRNA gene amplicon sequence variants (ASVs) that aligned within the Proteobacteria (28% mean relative abundance) including classes Gamma- and Alphaproteobacteria, Patescibacteria (24%), Campylobacterota (13%), and Desulfobacterota (8%) (Supplementary Fig. S3). Microbial communities partly clustered according to the water types BrMarine, Transition, and Saline although the BrMarine boreholes KA3105A_3 and KA2051A01_5 communities clustered with Saline samples (Supplementary Fig. S4). A db-RDA using only unique samples and the same eight water chemistry parameters as for DOM revealed that the water chemistry explained almost 92% of the variability (9999 permutations, *F* = 1.40, *n* = 10) of the microbial community composition. However, this relationship was not statistically significant, likely due to the low number of samples used in this study (*p* = 0.134, *n* = 10). Comparison of the DOM composition and the Äspö HRL groundwater microbial community compositions via Procrustes analysis resulted in an intermediate m^2^ of 0.65 (9999 permutations, *p* = 0.097) that indicated the datasets were not significantly related along main gradients of variability. Therefore, individual associations between microbial ASVs and DOM MFs were analyzed via network analysis based on proportionality. The MFs retained in the network were centered in van Krevelen space (Supplementary Fig. S5) but only comprised around 2% of the total relative sample intensities. Network MFs were overall of higher molecular weight and had higher heteroelements-to-carbon ratios than the original dataset (Table S2). In addition, the presumed lability of the MFs was higher as they contained less unsaturated hydrocarbon-type, more tannin-type, and less lignin-type MFs than the original dataset (Table S3). The composition of the ASVs found to be related to MFs also differed from the total community composition. DOM-related ASVs were dominated by Patescibacteria (12% mean relative abundance), Proteobacteria (9%), ‘unclassified’ taxon (5%), Desulfobacterota (4%), and Chloroflexota (3%). The resulting network consisted of 1617 nodes (1388 MFs and 229 ASVs), 2506 edges, 67 modules (Supplementary Data 1), and a network modularity of 0.89. Ten out of the 67 modules contained more than 50 nodes (3–23 ASV nodes and 54–259 MF nodes; Fig. 7, Tables S4, S5) that accounted for 63% of the whole network. The ten largest modules were comprised of MFs with distinct characteristics as reflected by varying H/C_MF_ and O/C_MF_ ratios ranging from 0.91 to 1.48 and 0.35 to 0.55 that formed clusters in specific areas of the van Krevelen diagram (Supplementary Fig. S5). Accordingly, the proportion of MFs assigned to specific compound classes differed between the modules (Table S4) with most MFs classified as lignin-like (ranging from 38 to 74% of the nodes per module).

On the basis of within- and between- module connectivity, 12 connectors, 45 module hubs, and 1560 peripheral nodes were identified in the whole network (Supplementary Fig. S6). While most connectors were MFs except for ASV165 (‘unclassified’ taxon), all but one module hubs were ASVs. All connector MFs contained the heteroelements N, S, or P (CHON, CHOS, CHOP, and CHONS) and were mostly lignin-like of varying lability and molecular weight. Most connectors were positively and negatively related to several microbial ASVs from different phyla such as Patescibacteria, Desulfobacterota, and Chloroflexota. Of the microbial module hubs, 16 (36%) were classified as Patescibacteria and seven (16%) as Desulfobacterota (Fig. 7).

## Discussion

### Regional chromatography shapes the fracture water signatures

Selective interactions of organic substances, especially N-rich materials, with water, microbes, and mineral surfaces during groundwater transport was reported by Aiken^41^ and termed “regional chromatography”^19^. This concept has been applied to describe groundwater DOM compositions^18,42^ and explains the refractory signal (i.e., low MLB_w_, low C/N_MF_) and relatively low variability in the Äspö HRL fracture system where lignin-like MFs contributed >50% of the relative intensities in all water types. In addition, that the sixfold change of bulk DOC concentrations between Baltic Sea and the deepest groundwaters plus the increased DON in the Baltic Sea were not mirrored in the narrow variation in DOM molecular characteristics can partially be attributed to the analytical window inherent to the applied methods^43^. However, the higher deep groundwater solid-phase extraction efficiencies suggested they contained a lower proportion of poorly captured high molecular weight material, colloidal organic matter, or small monomeric compounds compared to the Baltic Sea water. Despite a considerable terrestrial fingerprint^31^, the Baltic Sea DOM carries freshly produced matter comprising e.g., proteins, amino acids, and carbohydrates^44^. These compounds are preferentially respired in the subsurface water column and may adsorb to sediment grains leading to the strongly terrigenous DOM signature entering the deep fractures. In line with the changes in DOM composition with depth and groundwater type from the Baltic Sea downwards, Äspö HRL groundwater microbial communities supported by the available DOM also considerably differ from the overlying Baltic Sea water, suggesting species sorting occurred prior to the infiltrated waters reaching the Äspö HRL boreholes^45^. Thus, the existence of a Fennoscandian Shield core microbial community adapted to the oligotrophic subsurface^32^ may be at least partially due to the low variability and recalcitrant nature of the DOM in the different Äspö HRL groundwaters.

### Mixing dominates DOM signatures of waters from different origins

Groundwaters carrying >1 mg L^−1^ organic carbon are commonly located close to organic-rich sediments or receive organic-rich recharge^46^. The shallow BrMarine1 and BrMarine2 groundwaters with high surface connectivity exhibited large contributions of lignin- and tannin-like MFs of high aromaticity, similar to the high humic and fulvic acid concentrations from soil carbon of aromatic and (highly) unsaturated molecular classes detected in shallow soil-derived DOM^12,20,21^ and the Baltic Sea^31^. Toward the old Saline-type waters, DOC concentration, molecular mass and aromaticity, as well as O/C_MF_ and P/C_MF_ ratios consistently followed the dominant mixing process. The intrusion of terrigenous DOM into deep aquifers may confound previous interpretations of a largely chemolithoautotrophic origin of DOC in other systems^26,47^.

The Äspö HRL is built in Paleoproterozoic granitoids with fracture surfaces carrying calcite, chlorite, pyrite, and clay minerals^48,49^. Adsorption on surfaces or formation of insoluble complexes with hydrolyzing metals, especially Fe and Al, preferentially removes aromatic and lignin-like DOM moieties from solution^50,51^. The relatively high aromaticity of the BrMarine and partially the Transition-type DOM together with the high DOC concentrations indicated saturated surfaces and/or sufficient recharge^29^. Overall, that more than two thirds of the variability in DOM composition was explained by water chemistry parameters confirmed mixing of different source waters as the main driver of water compositions in the deep bedrock. The remaining variability points toward additional abiotic or biotic processes including sulfurization, sorption, condensation, or microbial uptake and reworking^6^ shaping DOM composition in Fennoscandian Shield groundwaters.

### Evidence for abiotic and biotic sulfurization of DOM

The high sulfate concentration in Saline waters and two Transition-type boreholes originated from Baltic Sea input and gypsum dissolution and were consistent with previous investigations in a nearby fracture system^52,53^. Microbial community data indicate the presence (Supplementary Fig. S3) and activity of autotrophic and heterotrophic sulfate reducing bacteria in Äspö HRL groundwaters^34,54^ that is not reflected in the alkalinity due to subsequent CO_2_ uptake in reciprocal microbial partnerships with acetogens, methanogens and fermenters^32,55,56^. In sulfidic sediments, organic matter reacts with reduced sulfur species potentially from sulfate reduction forming organic sulfur compounds as recently shown for DOM experimentally and in anoxic porewaters^15,16^, increasing organic matter preservation at low temperatures^17^. At Äspö HRL, the DOM S/C_MF_ ratio was elevated in all anoxic groundwaters (S/C_MF_ = 0.007; Supplementary Table S1) in comparison to Baltic Sea water (S/C_MF_ = 0.003). The S-content of the SPE-DOM (count of S-containing MFs or S/C_MF_ ratio) correlated to the relative contribution of the AbioS index indicative of abiotic sulfurization^40^, while the double bond equivalent (DBE) of S-containing MFs of the groundwaters was on average one unit lower than that of CHO-only MFs. Together with a negative relationship of S-containing and CHO-only contributions to sample compositions, this could indicate the formation of new DOC compounds through bisulfide addition^16^. However, the deviation in DBE was even higher at around two DBE for the oxic Baltic Sea samples, where a different mechanism must take place.

Sulfurization, manifested in increased S/C_MF_ ratios, of organic matter is thought to enhance preservation^17^. It may therefore seem counterintuitive that a higher S/C_MF_ ratio characterized DOM in the old Saline-type DOM that overall also comprised DOM of higher lability as indicated by the MLB_w_. Notably, on average, MFs in the dataset carrying at least one S (N) atom contributed approximately 13% (17%) of the total sample intensities. Separating relative contributions included in the MLB_w_ (H/C_MF_ > 1.5), CHO MFs contributed 65 ± 5% followed by S- (29 ± 4%) and N-containing (5 ± 2%) MFs. S-containing MFs thus contributed disproportionally highly to the labile DOM pool, especially compared to the often bioavailable N-containing compounds. The abiotic sulfurization process is not selective in regard to saturation, aromaticity, degree of oxidation, or heteroelement content of the precursor compound, and bisulfide nucleophilic addition shifts the MF toward higher H/C_MF_ ratios^15,16^. Furthermore, sulfur-containing metabolites produced though bacterial assimilatory S-reduction likely contributed to the DOS pool^57^. Hence, abiotic sulfurization of the subsurface DOM was an important process in the Äspö HRL fracture waters, but sulfur integration in DOM not only acted as a stabilizer but also increased the assumed lability of the DOM (% MLB_w_), an ambiguous role that needs further investigation in this system.

### Microbial imprint in old saline fracture waters

Microbial remineralization, transformation, and production affected the deep groundwater DOM compositions in addition to the dominant mixing signature. The microbial imprint was not only reflected in the old Saline DOM fingerprints, but also in the carbon isotopic composition. δ^13^C-DIC carbon isotopic values of −0.7 to −18.5‰ decreased with conductivity and represented recharging groundwaters with BrMarine influence, terrigenous organic matter oxidation and dissolution of calcite from different generations with only minor contributions of extremely depleted DIC derived from anaerobic oxidation of thermogenic (δ^13^C −30 to −50‰) or biogenic methane (δ^13^C −60 to −90‰) present at low concentrations in the fracture waters^38,53^. δ^13^C-DOC correlated with δ^13^C-DIC, but isotopic compositions were in a narrow range from −23.7 to −28.7‰ that were close the values reported for boreal forest soil (−24 to −29‰^58^), the dominant river DOM source in the region. In addition, the Δ^13^C_(DIC-DOC)_ values were mostly higher than the range of measured fractionation factors for the reverse tricarboxylic acid cycle (−2 to −12‰), emphasizing that CO_2_ fixation was not the main driver of DOC production as suggested for the hot Costa Rican backarc system^47^. Degradation by microbes typically lowers the δ^13^C of the remaining DOC pool^59^ and the most depleted δ^13^C-DOC value of −28.6‰ were found for the old saline DOM, indicating a stronger imprint in these waters to which a minor contribution of higher-plant derived material was previously attributed^60^. Usually, higher H/C_MF_ ratios or lower aromaticity of DOM are associated with younger DOM that is potentially more accessible to microbial utilization^61–63^, opposite to what was observed at the Äspö HRL. Here, in conjunction with the low AI_mod_, low molecular mass, and high H/C_MF_ ratios, permanent reworking and release of an aged but apparently labile organic matter fraction through the resident microbes was likely in the Saline-type waters^10^. In addition, the MFs unique to the deep Saline DOM were enriched in low-O/C_MF_ and high-H/C_MF_ MFs (Supplementary Fig. S1). These contrasting trends with ageing were recently attributed to the different oxygen availability and photochemical processing in oxic aquatic versus anoxic, deep systems where photodegradable compounds and aerobically biolabile formulas can accumulate^10^. In agreement with this and the DOM signature of microbial reworking at Äspö HRL, ref. 26 describe a deep groundwater DOM comprised of aliphatics, unsaturated, and lignin-like compounds also containing low molecular weight organic acids and minor contribution aromatic compounds likely of microbial origin in a deep South African fracture zone. These MFs were presumably either released by the resident microbial community through metabolic activity or after senescence, or indicated that allochthonous DOM transported in the aquifer had undergone methylation processes under anoxic conditions^64^. Possible methylation was supported by the higher average molecular weight of the MFs unique to Saline-type DOM (475 ± 26 Da) compared to the average molecular weight of all samples (397 ± 5 Da). Methane in the deep crystalline bedrock can originate from abiotic (e.g., Fischer-Tropsch) or biotic processes via methanogenesis^65^, but methanotrophic contribution to the DOM pool was low at Äspö according to the stable isotopic composition of DOC.

With ongoing microbial degradation of the DOM, N- and P-containing molecules are often preferentially utilized by heterotrophic bacteria^13,66^. However, these studies deal with DOM originating at least partially from recent primary production, whereas the DOM examined in the anoxic Fennoscandian Shield fractures had a terrigenous signature and had cycled through the subsurface bedrock fractures for long timescales. The fundamentally different source DOM quality and environment can entail that the opposite trend was found for the P/C_MF_ ratio with increasing age in the Äspö HRL fracture waters, and no consistent change in N/C_MF_ ratio was observed. Although terrestrial organic matter is generally depleted in nitrogen compared to marine organics, both the DON and DOC concentrations were lower in Transition- and Saline-type waters compared to the more recent BrMarine with no significant differences in bulk C/N ratios. The nitrogen-containing DOM may still be rapidly cycled in the environment limited in soluble nitrogen and phosphorus^67^, keeping the concentrations measured at discrete time points low. The increase in P/C_MF_ ratio was not easily explained and literature including DOP on MF level analysis is scarce. Phosphate was depleted in BrMarine2, Transition, and Saline waters, such that DOP comprises a potential P-source for microbial life rather than being released to the DOM pool. Yet, with the increase in P/C_MF_ ratio from Baltic Sea to Saline waters, also the number of P-containing MFs increased from around 50 to 150, pointing toward new production and no P-limitation of the subsurface microbes.

The possible reasons why the microbial imprint was primarily observed in the deep Saline-type DOM were threefold: (i) the DOC concentration was the lowest such that minor removal or dilution of terrigenous organics resulted in a visible microbial imprint in the relative abundance spectra; (ii) microbial adaptation to the refractory C sources takes time and thus only occurs in the oldest waters; or (iii) more accessible energy sources were available in other fracture waters. Previous studies show that the deep biosphere microbial community is viable and active, rapidly recycling dead cells as substrate^68^. Reworking of DOM due to microbial reuse of necromass adds to the labile characteristics of Saline-type DOM. In addition, carbon dioxide and hydrogen geogas-driven microbial metabolism may leave little imprint in the DOM and the fast uptake of products of this metabolism by other microbes further diminishes the detectable imprint^34^. However, all DOM fingerprints included in this study shared more than 85% of the MFs relative intensity, so that the proportion of the DOM responsible for the labile signature in the deep Saline waters was small in comparison to the recalcitrant, terrigenous signature common to all groundwaters. This potentially explains the slow growth rates and “stop-and-start” cell replication processes that are suggested to be activated or deactivated as and when carbon and energy sources are intermittently available to the deep fracture water microbial community^32^.

### Links between specific molecules and microbes

While both DOM and microbial community composition were to certain degrees linked to water type, overall compositions were not significantly related to each other. Nevertheless, network analysis revealed potential connections between DOM MFs and microbial ASVs in the form of correlations that require experimental evidence for confirmation. While microbial network ASVs accounted for around 37% of the total relative abundance, the network MFs only comprised around 2% of the total relative intensities. Considering that MFs in the network presumably represented compounds that were either produced or consumed by microorganisms, a low cumulative relative intensity of the respective MFs would be expected. In contrast, highly abundant MFs were more likely compounds of low bioavailability that accumulate in the groundwaters. This was mirrored in the higher lability (MLB_w_) of the network MFs and in line with previous studies reporting major proportions of refractory DOM in aquatic systems^18^. Most ASV nodes were affiliated with the phylum Patescibacteria that also constituted more than one-third of the module hubs that were highly connected and proposed to be keystone taxa^69^. These module hub Patescibacteria belonged to the classes Paceibacteria, ABY1, and Gracilibacteria, which were also present in a shallow groundwater system^70^ and are suggested to be prevalent in deep groundwaters by their ease of mobilization from soil coupled to their adaptation to low energy conditions^71^. Patescibacteria were not linked with specific DOM MFs, but with MFs of varying characteristics and all compound classes. In addition, they mostly co-occurred with either other Patescibacteria ASVs or ASVs affiliated with Chloroflexota, Dusulfobacterota, and ‘Unclassified’ phyla in the same module. This indicated coupling of the production and consumption of DOM compounds via a symbiotic host-associated lifestyle^72^. The coupled breakdown of complex DOM compounds was also supported by connector MFs, representing nodes that connect different modules (e.g., modules 3, 9, and 11) and therefore might reflect key intermediate compounds during microbial DOM degradation^69^. These connector MFs were positively and negatively linked to various microbial taxa, which might represent potential producers and consumers, respectively. Members of the phylum Desulfobacterota were also abundant in the network with module hub ASVs belonging to Desulfocapsa, Desulfatiglans, Desulfobacula, and unclassified genera of the order Syntrophales. Characterized members of the Desulfatiglans and Desulfobacula are heterotrophs and able to degrade aromatic compounds like phenol^73^, thus potentially being capable of degrading complex aromatic compounds like tannins and lignins in line with links between these two genera and mostly lignin-like MFs in the network (e.g., ASVs 218, 232, and 233; Fig. 7).

The small percentage of retained MFs suggested that although DOM was present, the large majority was resistant to microbial degradation. This supported the extremely long cell turnover times that have been calculated in the 100–1000 s of years for the deep marine biosphere^74^ and that the microbial community in terrestrial deep Saline waters in particular have been described as in “metabolic standby”^56^.

Organic carbon has a range of reactivities that are determined by the nature of the organic compounds, along with the biological, geochemical, and physical attributes of the environment^75^. The carbon advectively transported from Baltic Sea, meteoric, and soil sources into the deep continental bedrock fractures was mainly of terrigenous origin. The terrigenous DOM signature was retained and recalcitrant to degradation, potentially leading to the assembly of a core deep biosphere microbial community composition, supported by the low percentage of MFs associated to the community with Patescibacteria and Desulfobacterota as possible keystone taxa. Thus, deep Fennoscandian Shield microbes likely survive on the small pool of available molecules, such as the geogases carbon dioxide and methane plus the consumption of necromass as imprinted in the oldest, saline waters. This further supports the suggested “metabolic standby” of the communities, extremely long cell turnover rates, and “stop-and-start” replication processes. Future degradation experiments of the deep groundwater DOM under in situ conditions will aid to precisely disentangle mechanisms of subsurface DOM processing and its rates.

## Methods

### Sampling and water chemistry

In total, 13 packed-off borehole sections at depths ranging from 170 m to 507 mbsl were sampled for groundwater chemical components and isotopes (Fig. 1^76^) at the Äspö HRL on Äspö Island in the Misterhult archipelago in Kalmar County, Sweden. Since the tunnels were constructed between 1990 and 1995, contamination from excavation has been minimized although the boreholes have been instrumented for varying times that has introduced materials into the deep groundwaters. Samples from boreholes SA1229A_1, KA3105A_3, SA1730A_1, KA3600F_2, and SA2600A_1 were taken in November 2018 (batch 1) followed by all other samples in May 2019 (batch 2; Tables 1 and S1). The boreholes were chosen to cover relatively recent brackish-marine to pre-Holocene, saline waters. In addition, two surface Baltic Sea locations were sampled: one coastal site from Borholmsfjärden in the vicinity of the Äspö HRL (surface water, N 57°25.296’, E 16°39.583’) and one offshore sample at the LMO located 11 km offshore Kårehamn, Öland (2 m water depth, N 56°55.854’, E 17°3.642’).

Hydrochemical variables were determined in the ISO 17025 rated Äspö HRL chemical laboratory as follows: pH was determined potentiometrically (±0.10 pH units), Cl^−^ concentrations were determined by potentiometric titration (0.1 M AgNO_3_) with an analytical uncertainty of 6% (Swedish Standard 028136 edition 1), and SO_4_^2−^ concentrations were determined using ion chromatography (DIN EN ISO 10 304-1: 2009) with an analytical uncertainty of 12% (4.5–70 mg L^−1^) and 31% (0.5–4.5 mg L^−1^). Dissolved Fe^2+^ and total iron (Fe_total_) concentrations were determined immediately after sampling using a spectrophotometer (UVPC2401; Shimadzu, Kyoto Japan) and a modified ferrozine method^77^ with an analytical uncertainly of ±0.005% (0.02–0.05 mg L^−1^), 8% (0.05–1 mg L^−1^), and 13% (1–3 mg L^−1^) depending on concentration range. Oxygen concentrations could not be reliably measured due to the setup installed to obtain water from the boreholes, but Fe^2+^ concentrations equal to the Fe_total_ indicated anoxic conditions in all samples except those from the Baltic Sea. HS^−^ concentrations were determined with a spectrophotometer (as above) with an analytical uncertainty of 32% (Swedish Standard SIS 02 81 15). Concentrations of Ca, K, Mg, and Na cations were determined with inductively coupled plasma atomic emission spectroscopy at ALS Scandinavia AB in Luleå (Sweden) with an analytical uncertainty of 12%. Analysis of the water ^18^O/^16^O and ^2^H/^1^H ratio (per mil deviation from Standard Mean Ocean Water, SMOW) were determined by laser spectrometry (Los Gatos Research; Triple-Liquid Water Isotope Analyzer) with an analytical uncertainly of ±0.25 and ±1.5 unit, respectively. δ^13^C- and pMC-DIC and DOC were determined on whole water samples at the Ångström Laboratory in Uppsala (Sweden) using Accelerator Mass Spectrometry, with an analytical uncertainty of 0.3‰ PDB for δ^13^C and 0.2–0.5 pMC.

### Dissolved organic matter

All glassware used for sampling was pre-combusted (4 h, 400 °C) or thoroughly soaked in ultrapure water at pH 2 and rinsed with sample before use. Samples for DOM quantification and molecular characterization were filtered (Durapore PVDF 0.22 µm) and acidified to pH 2 with HCl before shipping to the University of Oldenburg (Germany). DOC and TDN were quantified from duplicate subsamples using a Shimadzu TOC-L with an ASI-L autosampler with concentrations determined via an L-arginine standard curve. Then, ~1 L of each sample was extracted onto 1 g solid-phase extraction columns (Bond Elut PPL, Agilent) and eluted with 6 mL methanol (ULC grade). DON was calculated from TDN − (nitrate + nitrite + ammonium). Extract DOC concentrations were determined by drying an aliquot of the extract and re-dissolving it in ultrapure water at pH 2. The extracts were then stored in the dark at −20 °C until further analysis. Stable carbon isotope composition of solid-phase extracted DOM (δ^13^C-SPE-DOC) was analyzed to better characterize the analytical window of the SPE method. Aliquots containing ~10 µmol C were dried in doubled Sn caps (IVA, Germany) and analyzed using an Elemental Analyzer (Thermo Scientific Flash) coupled to a continuous‐flow isotope ratio mass spectrometer (Thermo Scientific Delta V) via an Advantage Conflo IV interface. Values were corrected with procedural blanks, using the dried extracts from ultrapure water processing in doubled Sn caps. Technical replicates deviated <0.3‰, accuracy was better than 0.2‰.

Fourier-transform ion cyclotron resonance mass spectrometry (FT-ICR-MS) analysis was performed on a 15 T Solarix (Bruker Daltonics, Billerica, MA, USA, equipped with a ParaCell). Samples were diluted to yield a concentration of ~5 ppm in ultrapure water and methanol 50:50 (vol/vol). This dilution was filtered through pre-cleaned 0.2 µm polycarbonate syringe filters before analysis performed in random order. Electrospray ionization in negative mode (Bruker Apollo II) was done at 200 °C and the capillary voltage was set to 4.5 kV. The sample was injected at a flow rate of 120 µL h^−1^, the accumulation time was set to 0.05 s, and 300 scans were co-added for each spectrum in a mass range of 92–2000 Da. Batch 1 samples were analyzed once while batch 2 samples were run once when replicate extractions existed and twice from the same extract if only one extraction existed. Each spectrum was internally calibrated with lists of known masses. Mass spectra were exported from the Bruker Data Analysis software at a signal-to-noise ratio of 0 and MFs were assigned using the ICBM Ocean tool^78^. All analyses (batch 1 and 2) were processed together. The method detection limit was set to 3. Junction of mass lists along *m/z* was performed via fast join at a tolerance of 0.5 ppm while standard smooth and additional isotope tolerance was 10‰. Singlet peaks occurring only once in the dataset were removed, then MF attribution was done with a tolerance of 0.5 ppm in the range *m/z* 0–1000 within the limits C_1–100_H_1–100_O_0–70_N_0–4_S_0–2_P_0–1_. For isotopic verification of MF attributions, up to one ^13^C, ^15^N, and ^34^S isotopologue was allowed. Additional tolerance using quantile-based isotope ratio tolerances was set to 1000‰ and isotope ratio mismatches for peaks below the method detection limit <5 were permitted. MF suggestions with >3 heteroelements (except N_4_) and four common contaminant MFs were removed (C_16_H_32_O_2_, C_12_H_26_O_4_S, C_17_H_28_O_3_S, C_10_H_15_NO_2_S; likely NBBS from tubing) were removed from all samples. Before statistical analysis, relative intensities were normalized to the sum of intensities per sample and the highest minimum between all samples was set as detection limit and all peaks with lower normalized relative abundance were set to 0 to improve comparability between spectra. Furthermore, MFs were only retained in the dataset if they occurred at least three times across all samples. Molecular indices indicating lability through relative contribution of MFs above the molecular lability boundary of H/C_MF_ = 1.5, MLB_w_^79^, and aromaticity as modified aromaticity index, AI_mod_^80^, were calculated. MFs were assigned to molecular categories within the boundaries described by ref. 81 as unsaturated oxygen-poor (0 < O/C ≤ 0.29, 1.6 ≤ H/C ≤ 2.5), unsaturated (0.29 < O/C ≤ 0.6, 1.5 ≤ H/C ≤ 2.5), amino sugar- and carbohydrate-like (0.6 ≤ O/C ≤ 1.2, 1.5 ≤ H/C ≤ 2.5), unsaturated hydrocarbon-like (0 < O/C ≤ 0.29, 1 ≤ H/C ≤ 1.6), condensed aromatics (0 ≤ O/C ≤ 0.4, 0 ≤ H/C ≤ 0.7), lignin-like (0.29 < O/C ≤ 0.65, 0.7 < H/C < 1.5), and tannin-like (0.65 < O/C ≤ 1.2, 0.5 ≤ H/C < 1.5). MFs that could not be assigned to a class and MFs assigned to multiple categories were designated as unclassified. Elemental ratios calculated from FT-ICR-MS formula assignments were weighted by peak intensities and denoted throughout the manuscript via subscript MF (e.g., S/C_MF_).

### Microbial community composition

The Äspö HRL microbial community data used in this study was sampled in spring 2017 and published by ref. 33. While the groundwaters were sampled for microbial community analysis prior to the DOM sampling, studies at the Äspö HRL show that the water chemistry is relatively uniform and the microbial community’s rRNA and mRNA based composition and activities are stable over several years^55^. Microbial communities and the corresponding water chemistry data were sampled for boreholes SA1229A_1, KA2051A01_5, KA2511A_5, KA3105A_3, KA3600F_2, KA3385A_1, SA2600A_1, KA1755A_3, KA2862A_1, and SA1730A_1, whereas no data was available for the boreholes KA2865A01_1, KA3510A_2, and HA2780A_1. For a detailed description on sampling and sample processing refer to ref. 33. Briefly, planktonic microbial communities in Äspö HRL groundwaters were sampled in triplicates by connecting high-pressure filter holders (Millipore) to the borehole. Cells were captured on 0.1 µm pore size membrane filters and immediately flash frozen in liquid nitrogen. DNA from filters was extracted (MO BIO PowerWater DNA isolation kit) and the 16 S rRNA gene was amplified with the bacterial primers 341 F and 805R^82^. High-throughput sequencing was done on the Illumina MiSeq platform at the Science for Life Laboratory (Sweden) according to ref. 34. For this study, the sequencing data were reanalyzed using the DADA2 software package^83^, 1.14.1) in R (R Core Team; version 3.6.0). After the filter and trim step, remaining primer sequences were removed with Cutadapt (ref. 84, version 2.3). ASVs were inferred from denoised reads and after merging read pairs, ASVs were classified using the GTDB database (ref. 85; release 89). Details of the Äspö HRL DNA concentrations are provided in ref. 33. Details of the DADA2 reprocessed data are provided in Table S2. Rarefaction curves indicated a sufficient sequencing depth to capture the microbial diversity (Supplementary Fig. S7).

### Statistical analyses

Three different datasets were used throughout the manuscript: (1) DOM composition was analyzed based on all available samples including technical and analytical replicates (*n* = 35). (2) Statistical analyses including DOM and water chemistry were based on a dataset containing mean spectra of DOM from the same borehole regardless of sampling date (*n* = 15). (3) For statistical analyses including DOM composition, hydrochemistry, and microbial community compositions, the number of samples was further reduced due to the availability of the sequencing data (*n* = 10). Relative intensities of spectra, relative abundances of microbial ASVs, and environmental parameters were averaged per borehole for the analyses including more than one of these datasets.

Bray Curtis dissimilarities were used to assess the overall dissimilarity of the DOM fingerprints and microbial community composition and major gradients of variability were visualized with non-metric multidimensional scaling (NMDS). The congruence of DOM molecular and microbial community composition was analyzed via Procrustes rotation. The impact of mixing on DOM and microbial community composition was assessed via distance-based redundancy analysis. These statistical analyses were performed using R (Version 4.0.3) and the vegan package^86^. Pairwise associations between microbial ASVs and DOM MFs were investigated via proportionality analysis to account for the compositional nature of both datasets^87^. For this, the overlapping datasets for DOM and microbial community composition were used (*n* = 10). MFs and ASVs were only considered for the analysis if they were present in at least five samples; low-abundant ASVs were removed (relative abundance < 0.1%); zeros were replaced by their respective minimum value/10 to account for the fact that the range of relative intensities and abundances covered several orders of magnitude; and each dataset was center log-ratio (clr) transformed separately. Proportionality measure ρ was then calculated for the combined ASV and MF data matrix (propr package). The selection of a cutoff for ρ was based on permutation of the false-discovery rate (FDR) for different ρ^88^. To control for a FDR < 5%, |ρ | ≥ 0.85 was chosen as cutoff for network construction. The resulting network retained a relationship between the relative abundances of 229 ASVs and relative intensities of 1388 MFs. Network visualization was done in Gephi (version 0.9.2^89^). Network modularity, module separation and roles of nodes were determined via the interdomain ecological network analysis pipeline (IDENAP; http://mem.rcees.ac.cn:8081^90^) with the fast greedy modularity optimization^91^. The potential ecological role of each node was assigned based on within-module connectivity (z) and among-module connectivity (P)^92^ and nodes were classified into four categories: peripheral nodes (*z* ≤ 2.5, *P* ≤ 0.62), connectors (*z* ≤ 2.5, *P* > 0.62), module hubs (*z* > 2.5, *P* ≥ 0.62), and network hubs (*z* > 2.5, *P* > 0.62)^69^.

## Supplementary information

Supplementary Information

Description of Additional Supplementary Files

Supplementary Data 1

## Data Availability

16 S rRNA gene reads are available at the NCBI Sequence Read Archive (SRA) with the Bioproject accession number PRJNA434543. FT-ICR-MS data and water chemistry generated in this study are available through the Pangaea database^93^.
