# Supplementary material for: Terrigenous dissolved organic matter persists in the energy-limited deep groundwaters of the Fennoscandian Shield
Source: Nat Commun. 2022 Aug 17;13:4837. doi: 10.1038/s41467-022-32457-z (PMC9385861; doi:10.1038/s41467-022-32457-z)
Supplement: Supplementary file 1 — Supplementary Information [file 41467_2022_32457_MOESM1_ESM.pdf]

Supplementary Information to:

Terrigenous dissolved organic matter persists in the energy-limited deep groundwaters of the Fennoscandian Shield

Helena Osterholz[*1], Stephanie Turner[2,3], Linda J. Alakangas[2,4], Eva-Lena Tullborg[5], Thorsten Dittmar[6,7], Birgitta E. Kalinowski[4], Mark Dopson[2]

[1]Marine Chemistry, Leibniz Institute for Baltic Sea Research Warnemünde, Rostock, Germany

[2]Ecology and Evolution in Microbial model Systems (EEMiS), Linnaeus University, Kalmar, Sweden

[3]Department of Forest Mycology and Plant Pathology, Swedish University of Agricultural Sciences, Uppsala, Sweden

[4]Swedish Nuclear Fuel and Waste Management Company, Äspö Hard Rock Laboratory, Oskarshamn, Sweden

[5]Terralogica AB, Gråbo, Sweden

[6]Marine Geochemistry, Institute for Chemistry and Biology of the Marine Environment, Carl von Ossietzky University, Oldenburg, Germany

[7]Helmholtz Institute for Functional Marine Biodiversity, Carl von Ossietzky University, Oldenburg, Germany

*corresponding author, Email: helena.osterholz@io-warnemuende.de

Supplementary Information

A. Supplementary Tables

Table S1: Additional water chemistry parameters of Baltic Sea water and boreholes.

Borehole/ Sampling Area	Water type (this study)	Date	Number of assigned MFs	*m/z*	O/C_{MF}	H/C_{MF}	N/C_{MF}	S/C_{MF}	P/C_{MF}	MLB_w-CHO	MLB_w-with S	AI_{mod}	AbioS peaks (% intensity)
		(mm-yy)											
**Baltic Sea water**													
LMO	Baltic Sea	04-19	7430±30	402±5	0.47±0.00	1.22±0.00	0.010±0.000	0.003±0.000	0.0001±0.0000	4.4±0.1	1.5±0.0	0.27±0.00	0.0000±0.0000
Äspö	Baltic Sea	04-19	7622±8	416±3	0.47±0.00	1.17±0.01	0.007±0.000	0.003±0.000	0.0000±0.0000	3.3±0.1	1.1±0.0	0.30±0.00	0.0000±0.0000
**Äspö HRL groundwaters**													
SA1229A_1	BrMarine1	11-18	8858±NA	412	0.46	1.18	0.011	0.008	0.0001	2.8	1.6	0.29	0.0004
		04-19	8692±166	402±2	0.45±0.01	1.19±0.01	0.011±0.000	0.008±0.000	0.0000±0.0000	3.3±0.3	1.4±0.1	0.29±0.00	0.0004±0.0000
KA2051A01_5	BrMarine1	03-19	7697±204	425±7	0.44±0.00	1.22±0.00	0.006±0.000	0.004±0.000	0.0001±0.0000	3.4±0.0	1.2±0.0	0.28±0.00	0.0000±0.0000
KA2511A_5	BrMarine1	03-19	8564±21	410±3	0.45±0.00	1.20±0.01	0.009±0.000	0.006±0.000	0.0001±0.0000	3.6±0.2	1.6±0.1	0.28±0.00	0.0002±0.0000
KA3105A_3	BrMarine2	11-18	7793	421	0.44	1.23	0.007	0.004	0.0001	3.8	1.4	0.27	0.0000
		03-19	7823±289	420±6	0.44±0.01	1.22±0.01	0.007±0.000	0.004±0.000	0.0001±0.0000	3.7±0.5	1.4±0.1	0.27±0.00	0.0000±0.0000
KA3600F_2	BrMarine2	11-18	8818	401	0.43	1.22	0.011	0.010	0.0002	3.9	2.4	0.28	0.0006
		03-19	8561±78	396±1	0.44±0.00	1.21±0.00	0.011±0.000	0.010±0.000	0.0001±0.0000	3.9±0.0	2.2±0.2	0.28±0.00	0.0006±0.0000
KA2865A01_1	Transition	03-19	7763±361	411±1	0.44±0.01	1.22±0.01	0.007±0.000	0.004±0.000	0.0002±0.0000	3.8±0.3	1.3±0.1	0.28±0.00	0.0001±0.0000
KA3385A_1	Transition	03-19	7477±292	397±1	0.42±0.01	1.26±0.01	0.008±0.001	0.006±0.001	0.0002±0.0001	5.2±0.6	1.9±0.3	0.26±0.00	0.0003±0.0000
KA3510A_2	Transition	03-19	7917±144	389±3	0.42±0.00	1.26±0.00	0.010±0.000	0.009±0.000	0.0003±0.0000	5.3±0.2	2.7±0.1	0.26±0.00	0.0006±0.0000
SA2600A_1	Saline	11-18	7935	398	0.43	1.24	0.009	0.007	0.0003	4.7	2.1	0.27	0.0003
		03-19	8124±144	402±3	0.43±0.00	1.24±0.00	0.009±0.000	0.007±0.000	0.0002±0.0000	4.5±0.0	2.2±0.1	0.27±0.00	0.0003±0.0000
HA2780A_1	Saline	03-19	7289±223	394±0	0.43±0.01	1.26±0.01	0.008±0.001	0.006±0.000	0.0002±0.0001	5.4±0.6	2.1±0.1	0.26±0.00	0.0002±0.0000
SA1730A_1	Saline	11-18	8014	399	0.43	1.24	0.010	0.008	0.0003	4.5	2.5	0.27	0.0006
		03-19	8127±227	404±2	0.44±0.00	1.23±0.00	0.009±0.000	0.007±0.000	0.0002±0.0000	4.2±0.1	2.3±0.1	0.27±0.00	0.0005±0.0000
KA1755A_3	Saline	03-19	7684±78	388±2	0.42±0.00	1.26±0.00	0.012±0.000	0.010±0.000	0.0003±0.0001	5.0±0.1	2.9±0.1	0.26±0.00	0.0008±0.0000
KA2862A_1	Saline	03-19	7839±19	399±2	0.43±0.00	1.25±0.00	0.010±0.000	0.007±0.000	0.0003±0.0000	4.6±0.2	2.4±0.0	0.26±0.00	0.0004±0.0000

Table S1 continued.

Borehole/ Sampling Area	Water type (this study)	Date	$\delta^{13}C$-DIC	$\delta^{13}C$-DOC	$\delta^{13}C$ -SPE-DOC	pMC_DIC	pMC_DOC
		(mm-yy)	(‰)	(‰)	(‰)	(%)	(%)
**Baltic Sea water**							
LMO	Baltic Sea	04-19	-0.7	-26.3	-27.7	100.1	100.4
Äspö	Baltic Sea	04-19	-2.9	-25.6	-28.2	105.6	99.2
**Äspö HRL groundwaters**							
SA1229A_1	BrMarine1	11-18	-5.3	-26.2	-26.9	78.4	84.7
		04-19	-5.3	-26.2	-26.6	81.7	86.5
KA2051A01_5	BrMarine1	03-19	-14.4	-26.9	-28.1	62.2	105.7
KA2511A_5	BrMarine1	03-19	-9.3	-25.8	-27.1	34.5	89.7
KA3105A_3	BrMarine2	11-18	-13.2	-27.5	-28.2	64.8	100.8
		03-19	-13.0	-26.6	-28.0	65.3	100.7
KA3600F_2	BrMarine2	11-18	-6.2	-25.3	-26.4	68.1	78.2
		03-19	-6.3	-24.0	-26.1	55.5	79.3
KA2865A01_1	Transition	03-19	-14.7	-26.5	-27.7	64.7	93.6
KA3385A_1	Transition	03-19	-8.0	-26.8	-27.0	46.6	68.4
KA3510A_2	Transition	03-19	-8.4	-25.8	-26.6	46.3	73.4
SA2600A_1	Saline	11-18	-17.3	-28.6	-27.3	41.1	73.0
		03-19	-13.2	-23.7	-27.3	38.6	73.0
HA2780A_1	Saline	03-19	-11.0	-27.8	-27.3	50.9	71.7
SA1730A_1	Saline	03-19	-14.9	-28.7	-27.4	38.4	69.7
		11-18	-18.5	-28.3	-27.4	38.9	68.3
KA1755A_3	Saline	03-19	-11.5	-26.6	-27.1	47.8	58.2
KA2862A_1	Saline	03-19	-11.2	-28.3	-27.4	43.2	71.1

Table S2: Details for 16S rRNA gene amplicon sequencing data processing. Details for three replicates per borehole are provided.

Sample (Borehole_replicate)	Read pairs (2 × 301 bp)	Read pairs after filtering	Read pairs after merging	Read pairs after chimera removal	Number of ASVs
**SA1229A-nit-T1**	126805	90144	69501	60228	769
**SA1229A-nit-T2**	310681	230223	179623	147331	1562
**SA1229A-nit-T3**	296671	218520	170843	137709	1304
**KA2051A01-5-1**	233329	150682	100907	64943	949
**KA2051A01-5-2**	180096	116417	82410	59616	892
**KA2051A01-5-3**	185862	127011	91932	61133	807
**KA2511A-5-1**	271334	188020	151587	117347	805
**KA2511A-5-2**	467106	326019	281738	189864	860
**KA2511A-5-3**	358758	250716	209746	124797	748
**KA3105A-3-1**	92757	63412	44422	34506	487
**KA3105A-3-2**	138999	97414	68583	49370	551
**KA3105A-3-3**	138664	98387	75692	59116	488
**KA3600F-2-1**	181389	123874	93873	72133	731
**KA3600F-2-2**	320223	218464	164118	105541	886
**KA3600F-2-3**	380297	261840	206648	117829	928
**KA3385A-nit-T1**	297445	216542	184849	121675	542
**KA3385A-nit-T2**	194555	140739	120976	95850	559
**KA3385A-nit-T3**	466187	342754	304491	196538	526
**SA2600A-1**	239919	170392	130606	97926	921
**SA2600A-2**	336792	239816	188177	125424	972
**SA2600A-3**	380877	270366	219192	152991	1060
**SA1730A-1**	66266	42468	28475	23768	399
**SA1730A-2**	101824	67812	50282	45894	684
**SA1730A-3**	151534	102669	77176	56274	673
**KA1755A-3-1**	566387	432452	377378	303959	1125
**KA1755A-3-2**	105063	75448	61262	54269	515
**KA1755A-3-3**	56708	35546	22933	20314	349
**KA2862A-1**	260018	188639	146844	108337	766
**KA2862A-2**	235214	170166	131967	93446	700
**KA2862A-3**	299168	216001	173189	136382	985

Table S3: DOM composition including (1) all MFs, (2) MFs submitted to network analysis, and (3) MFs included in the network. The given means are calculated from the averaged spectra included in the network analysis (n=10). Mean±sd are given, sd is standard deviation of all samples.

	all DOM	filtered DOM	network DOM
**Count overall**	12728	9610	1388
**Relative intensity (%)**	100	99±0.3	2.4±0.54
**Molecular mass (Da)**	405±11	403±11	508±36
**H/C_{MF}**	1.23±0.02	1.23±0.02	1.15±0.10
**O/C_{MF}**	0.44±0.01	0.44±0.01	0.49±0.03
**N/C_{MF}**	0.010±0.002	0.010±0.002	0.024±0.006
**S/C_{MF}**	0.008±0.003	0.008±0.002	0.019±0.009
**P/C_{MF}**	0.0002±0.0001	0.0001±0.0001	0.0048±0.0036
**AI_{mod}**	0.27±0.01	0.27±0.01	0.29±0.04
**Lability Index MLB_w**	6.5±1.4	6.5±1.3	10.9±7.5
**Unsaturated O-poor (%)**	1.1±0.1	0.4±0.1	0.7±0.8
**Unsaturated (%)**	10.3±0.3	7.4±1.4	8.7±5.8
**Amino sugar and carbohydrate-like (%)**	3.1±0.2	0.5±0.1	3.1±1.8
**Unsaturated hydrocarbon-like (%)**	11.1±0.3	7.3±0.9	5.2±2.7
**Condensed aromatics(%)**	4.4±0.1	0.6±0.2	3.2±1.1
**Lignin-like(%)**	53.0±1.4	79.5±1.1	58.1±3.7
**Tannin-like (%)**	8.3±0.3	2.6±0.9	14.5±6.1

Table S4: DOM composition of 10 modules containing >50 nodes. As the modules are contained in the network because they show some kind of gradient, we here show averages that are not weighted by MF relative intensities.

Module	6	3	2	18	5	20	1	4	11	9
MF count	259	134	95	74	71	67	58	58	56	54
Molecular mass (Da)	589±191	543±180	508±152	502±160	494±233	406±95	462±150	492±119	490±132	472±118
H/C_{MF}	1.09±0.23	1.25±0.39	1.05±0.29	1.01±0.23	1.26±0.31	1.13±0.24	0.97±0.27	1.48±0.27	1.27±0.49	0.91±0.27
O/C_{MF}	0.55±0.15	0.51±0.19	0.49±0.12	0.48±0.14	0.35±0.15	0.40±0.13	0.43±0.15	0.46±0.18	0.49±0.23	0.50±0.18
N/C_{MF}	0.013±0.030	0.044±0.070	0.028±0.043	0.038±0.051	0.030±0.052	0.041±0.048	0.034±0.048	0.023±0.058	0.042±0.073	0.025±0.037
S/C_{MF}	0.006±0.017	0.013±0.026	0.019±0.029	0.035±0.037	0.055±0.073	0.059±0.049	0.038±0.042	0.004±0.012	0.018±0.025	0.023±0.029
P/C_{MF}	0.0006±0.0074	0.0085±0.0186	0.0008±0.0079	0.0000±0.0000	0.0025±0.0111	0.0000±0.0000	0.0037±0.0123	0.0242±0.0264	0.0097±0.0215	0.0010±0.0072
AI_{mod}	0.30±0.16	0.25±0.20	0.35±0.22	0.35±0.19	0.25±0.21	0.30±0.19	0.40±0.21	0.13±0.12	0.28±0.26	0.42±0.23
Unsaturated O-poor (%)	0	1	0	0	1	0	0	2	0	0
Unsaturated (%)	2	10	4	1	17	6	5	34	16	2
Amino sugar and carbohydrate-like (%)	2	7	3	0	1	0	0	7	18	0
Unsaturated hydrocarbon-like (%)	1	4	1	3	20	13	5	7	5	2
Condensed aromatics(%)	2	1	5	4	6	0	9	0	11	7
Lignin-like(%)	68	66	73	74	48	72	57	48	38	46
Tannin-like (%)	22	5	3	7	3	1	5	2	2	20
unclassified	4	4	11	11	4	7	19	0	11	22
CHOS	14	22	32	41	34	45	34	7	32	30
CHNO	17	39	31	31	17	25	26	22	38	26
CHO	68	21	31	12	28	0	14	22	5	31
CHNOS	0	1	6	16	14	30	17	2	7	11
CHOP	1	15	1	0	1	0	7	47	18	2
CHOPS	0	3	0	0	0	0	2	0	0	0
CHNOP	0	0	0	0	4	0	0	0	0	0
CHN	0	0	0	0	1	0	0	0	0	0

Table S5: Taxonomic association of network ASVs. Microbial community composition of 10 modules containing >50 nodes as relative proportion of nodes assigned to the different phyla.

**Module**	**6**	**3**	**2**	**18**	**5**	**20**	**1**	**4**	**11**	**9**
**ASV count**	**7**	**14**	**8**	**10**	**7**	**3**	**23**	**6**	**8**	**3**
**Actinobacteriota**	14	7	0	0	0	0	0	0	0	0
**Bacteroidota**	0	7	0	0	0	0	4	0	0	0
**Caldatribacteriota**	0	7	0	0	0	0	0	33	13	0
**Campylobacterota**	0	0	0	0	0	0	4	17	13	0
**Chloroflexota**	0	7	0	0	0	0	22	17	0	0
**Desulfobacterota**	14	29	25	10	14	67	9	17	0	0
**Desulfobacterota_A**	0	0	13	0	0	0	0	0	0	0
**Firmicutes_A**	0	0	0	0	0	0	4	0	0	0
**Halobacterota**	0	7	0	0	0	0	0	0	0	0
**Margulisbacteria**	14	0	0	0	0	0	4	0	0	0
**Nanoarchaeota**	14	0	0	0	0	0	0	0	0	0
**Nitrospirota**	0	0	0	10	0	0	0	0	0	0
**Omnitrophota**	0	0	13	10	43	0	4	0	0	0
**Patescibacteria**	43	21	13	30	14	0	39	17	38	100
**Planctomycetota**	0	0	0	10	0	0	0	0	0	0
**Proteobacteria**	0	0	25	0	14	33	4	0	0	0
**Unclassified**	0	14	13	30	0	0	4	0	38	0
**Verrucomicrobiota**	0	0	0	0	14	0	0	0	0	0

B. Supplementary Figures

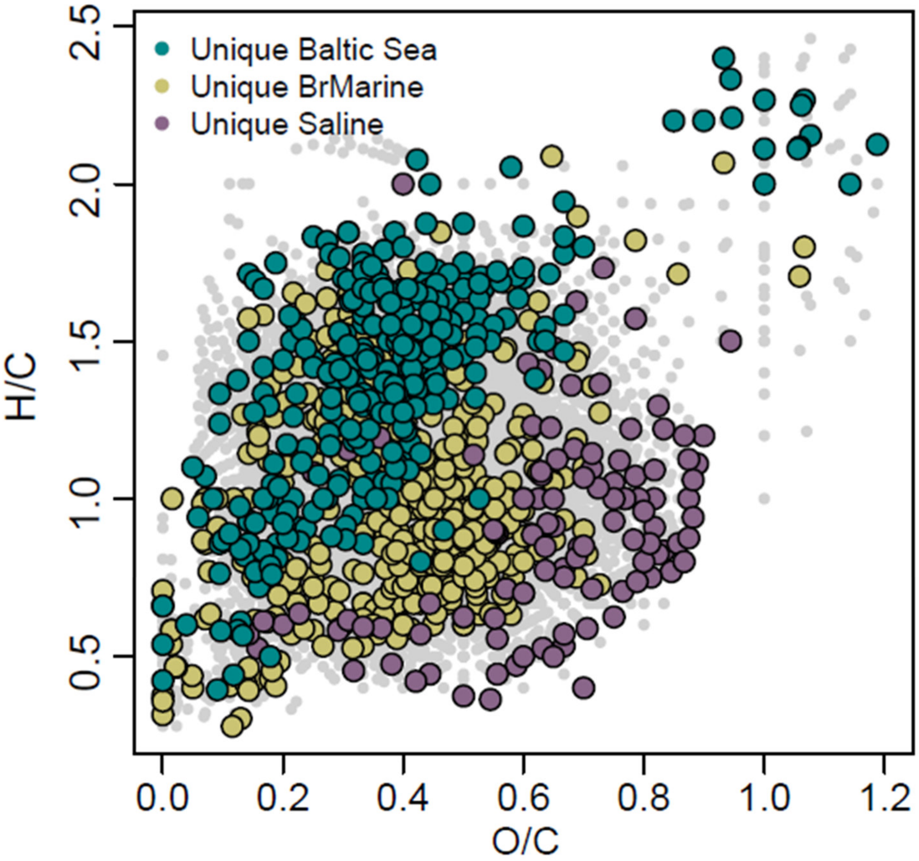

Figure S1: Van Krevelen diagram of unique MFs per water type. Diagram shows all MFs (grey), MFs only detected in Saline-type DOM (n=110), MFs detected only in Baltic Sea samples (n=309) and MFs only detected in samples of BrMarine origin (n=438). Transition-type DOM ignored for the definition of unique peaks as it was so widely distributed across compositions.

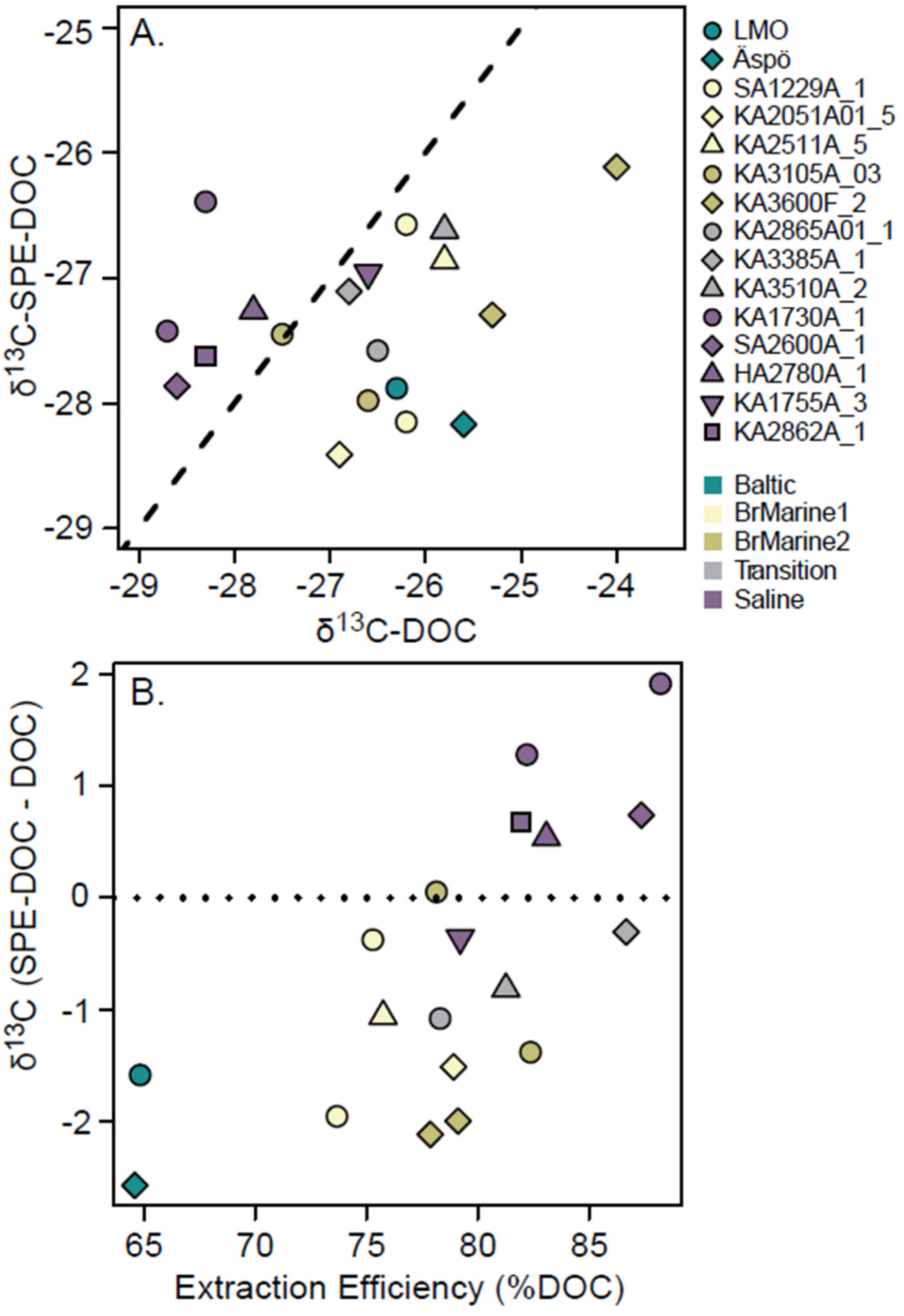

Figure S2: Stable carbon isotopic composition of carbon fractions. Isotopic composition of bulk δ^{13}C-DOC and extracted δ^{13}C-SPE-DOC organic carbon (A; R=0.38, p<0.05) and relationship to extraction efficiency (B). Black line in (A) is 1:1 line, dashed black line in (B) denotes no difference between bulk and extracted carbon isotopic compositions.

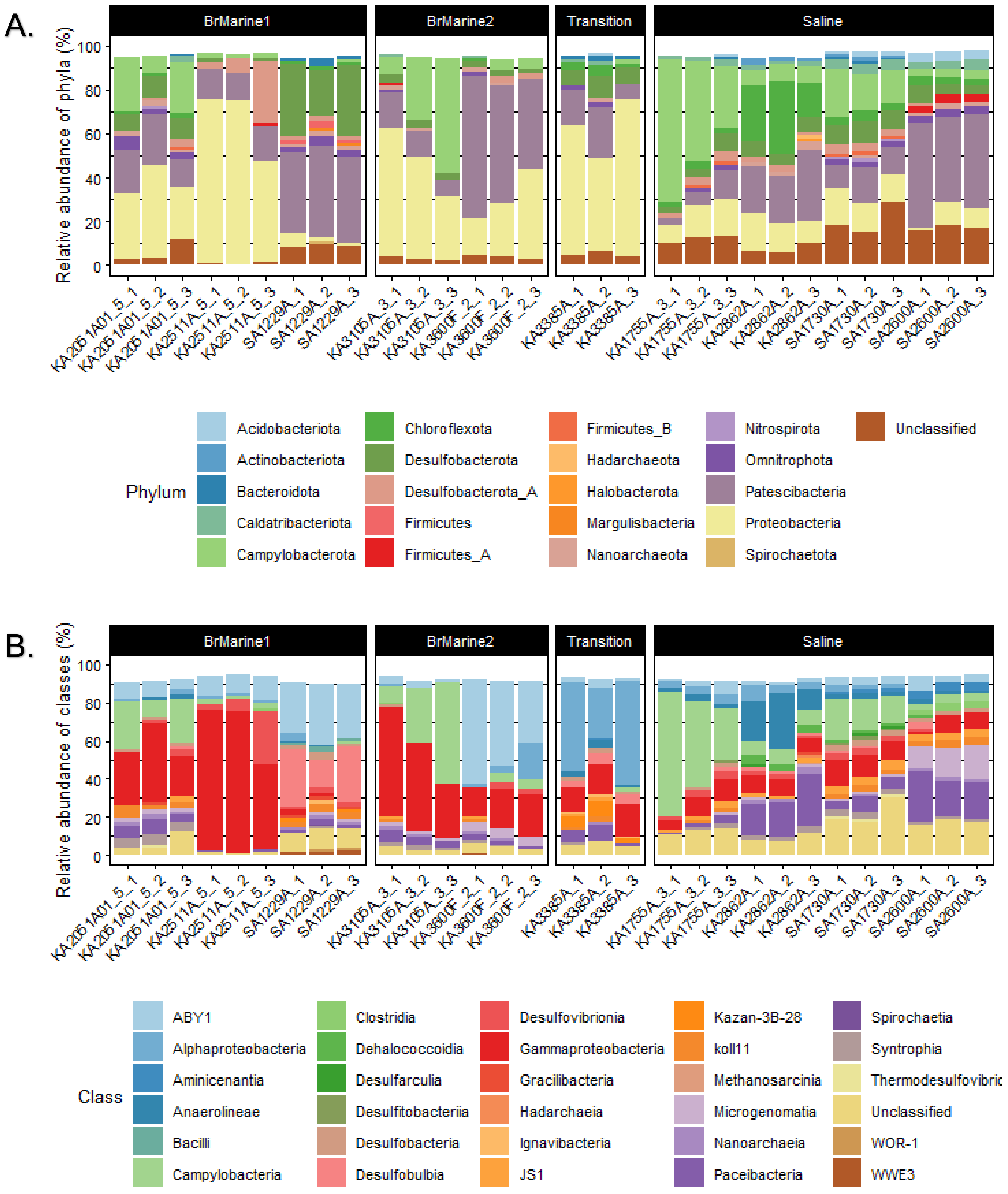

Figure S3: Microbial community composition in Äspö HRL groundwaters. Stacked bar graph of the microbial community composition based on the relative abundances (> 1%) of phyla (A) and classes (B). The remaining proportion to 100% includes low-abundant taxa of <1% of the relative abundance.

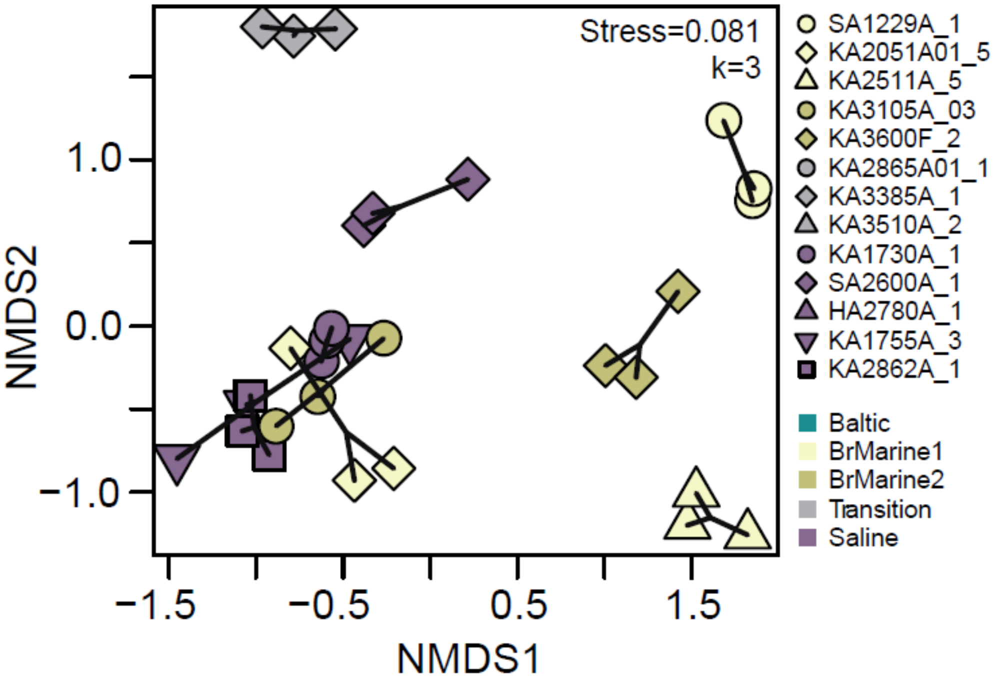

Figure S4: NMDS of microbial community composition in Äspö HRL groundwaters. The NMDS is based on ASV relative abundances of triplicate analyses per borehole. Black lines connect replicate samples.

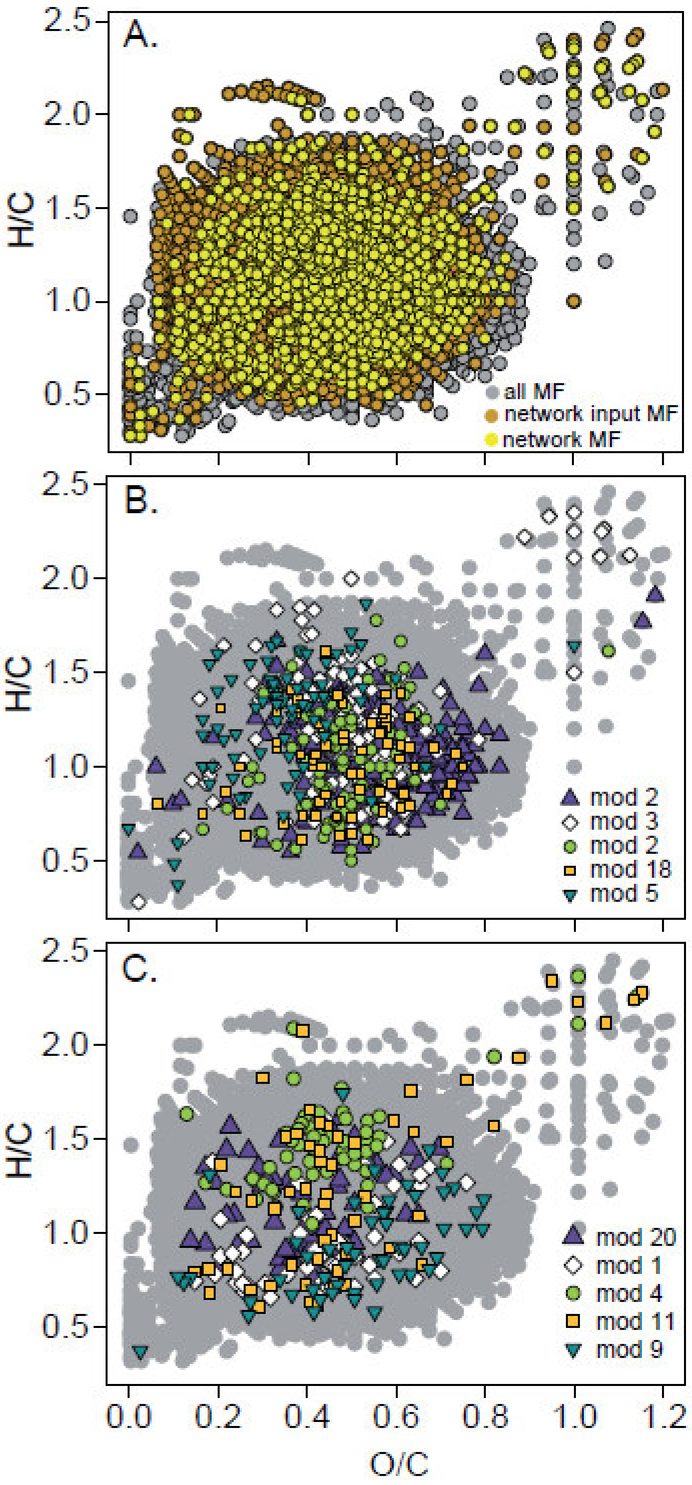

Figure S5: Van Krevelen diagrams of network MFs. Distributions of all MFs, MFs used in network calculation and MFs occurring in final network cover similar ranges (A). MFs in top 10 modules (mod) comprising >50 MFs (B,C).

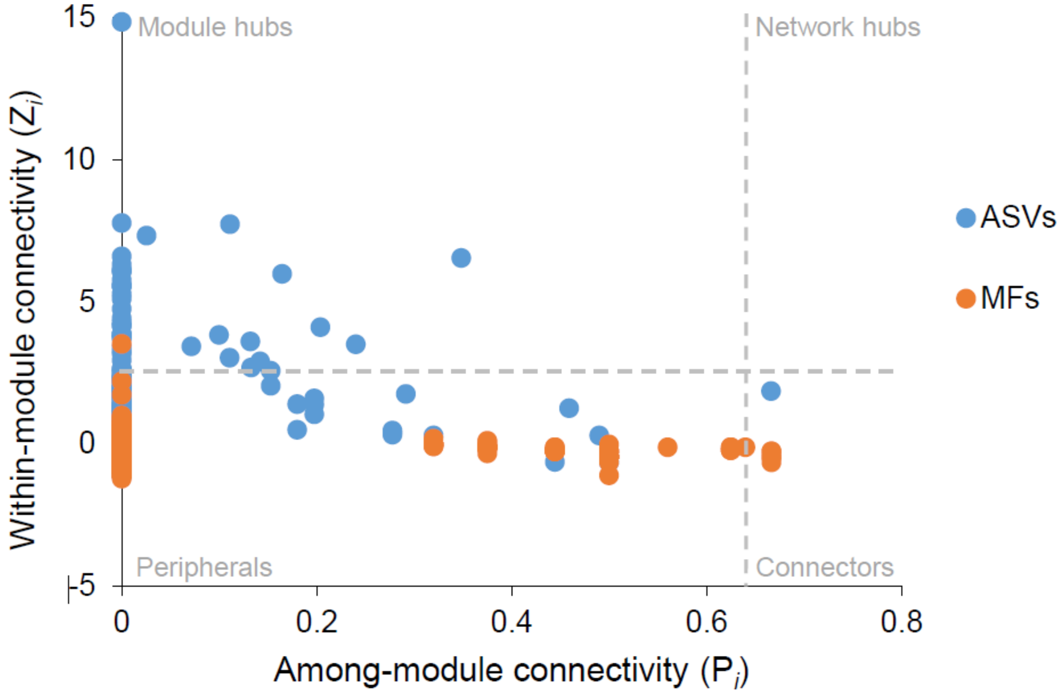

Figure S6: Potential roles of network nodes based on within-module and among-module connectivity. Orange circles represent DOM MFs and blue circles represent microbial ASVs.

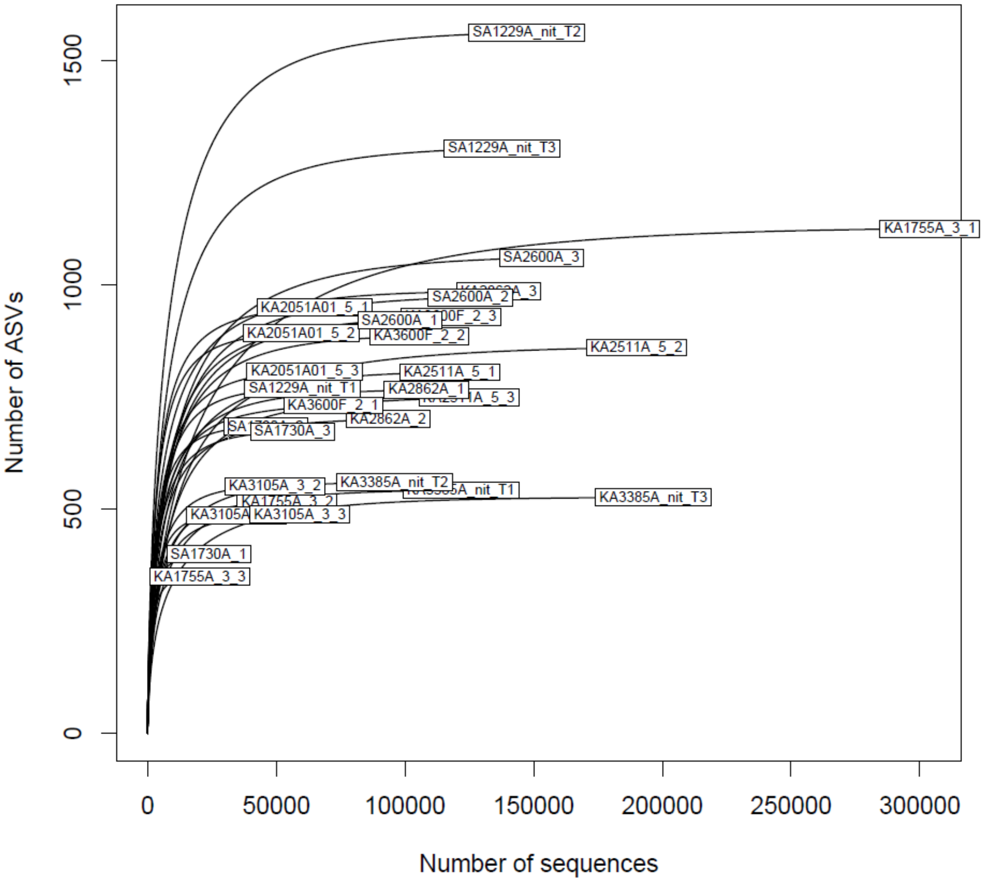

Figure S7: Rarefaction curve showing the sequencing depth for Äspö HRL groundwater samples.
