# Supplementary material for: Terrigenous dissolved organic matter persists in the energy-limited deep groundwaters of the Fennoscandian Shield
Source: Nat Commun. 2022 Aug 17;13:4837. doi: 10.1038/s41467-022-32457-z (PMC9385861; doi:10.1038/s41467-022-32457-z)
Supplement: Supplementary file 2 — Description of Additional Supplementary Files [file 41467_2022_32457_MOESM2_ESM.pdf]

Description of Additional Supplementary Files

File Name: Supplementary Data 1

Description: **Characteristics of network nodes.** The table includes module assignment and role of network nodes corresponding to Fig. 7. For ASVs, phylogenetic information is provided; for molecular formulas, m/z, H/C ratio, AImod, DBE, and compound class are included.
